# m^6^A methyltransferase KIAA1429 acts as an oncogenic factor in colorectal cancer by regulating SIRT1 in an m^6^A-dependent manner

**DOI:** 10.1038/s41420-022-00878-w

**Published:** 2022-02-25

**Authors:** Yuan Zhou, Zhengda Pei, Aizezi Maimaiti, Linyi Zheng, Zhongcheng Zhu, Mengxiang Tian, Zhongyi Zhou, Fengbo Tan, Qian Pei, Yuqiang Li, Wenxue Liu

**Affiliations:** 1 Department of General Surgery, Xiangya Hospital, Central South University, Changsha, China; 2 National Clinical Research Center for Geriatric Disorders, Xiangya Hospital, Central South University, Changsha, China; 3 Clinical College, Xiangnan University, Chenzhou, China; 4 Department of Cardiology, Xiangya Hospital, Central South University, Changsha, China; 5 Department of Rheumatology, Guangdong Provincial People’s Hospital, Guangdong Academy of Medical Sciences, Guangzhou, China

**Keywords:** Colorectal cancer, Tumour biomarkers

## Abstract

N6-methyladenosine (m6A) modifications of RNAs are involved in various aspects of colorectal carcinogenesis via regulation of mRNA stability, splicing, and translation. KIAA1429, an m6A methyltransferase, was found deregulated in multiple cancer types. However, its role in colorectal cancer remains elusive. By analyzing TCGA and GEPIA database, we found that KIAA1429 in colorectal cancer was highly expressed. In addition, we used immunohistochemistry, western blotting, and QRT-PCR to detect the expression of KIAA1429 in colorectal cancer samples and cell lines, and we found that KIAA1429 was overexpressed in colorectal cancer sample and cell line. Functionally, silencing of KIAA1429 by shRNA in colorectal cancer cell lines resulted in decreased cell proliferation, colony formation, and migration. On the contrary, overexpression of KIAA1429 increased cell proliferation, colony formation, and migration. Further mechanism analysis demonstrated that KIAA1429 increased the expression of SIRT1 via regulating its mRNA stability in an m6A-dependent manner. More importantly, in vivo experiment showed that depletion of KIAA1429 significantly inhibited colorectal tumor growth. In conclusion, our results suggested that the m6A methyltransferase KIAA1429 promotes the growth and motility of colorectal cancer and could be a potent therapeutic target.

## Introduction

Colorectal cancer, a widespread malignant tumor, is the third most frequent cancer and one of the leading causes of cancer-related death globally [1]. Despite advances in therapeutic methods, such as surgical resection combined with chemotherapy and radiotherapy, the progress in colorectal cancer treatment remains sluggish. Approximately 40–50% of newly diagnosed patients developed metastatic tumors, and small fraction of these patients can undergo curative resection and the overall survival of the patients with metastatic tumors reaches only 30 months [2]. Surgery and radiotherapy are widely used in clinics. However, it has been identified that both surgery and radiotherapy triggered undesirable metastasis in certain cases, which limits their use [3–7]. Thus, chemotherapy drugs are widely used based on their inhibitory effect on the growth and induction of apoptosis in cancer cells. Numerous studies are attempting to investigate the underlying mechanism and develop novel therapeutic targets to improve the therapy.

RNA modification is an important part of biological processes, and *N*6-Methyl- adenosine (m^6^A) is one of the most widely distributed RNA modifications in eukaryotes [8, 9]. m^6^A is a reversible and dynamic process regulated by the so-called ‘writer’ (methyltransferase complex), ‘eraser’ (demethylases), and ‘reader’. In m^6^A ‘writers’ complex, the methyltransferase-like 3 (METTL3) and METTL14 form a heterodimer and interact with Wilms tumour1-associated protein (WTAP) in the nucleus [10]. Recently, vir-like m6A methyltransferase associated (VIRMA; also known as KIAA1429), RNA-binding motif protein 15 (RBM15), zinc finger CCCH-type containing protein 13 (Zc3h13), and METTL16 were also found to be the components of the methyltransferase complex [11–14]. m^6^A modification has been shown to play essential roles in various bioprocesses, including tissue development, stem cell formation and differentiation, heat shock response control, and circadian clock control [15–17]. In addition, dysregulation of m^6^A modification was also found to play distinct roles in multiple cancers. KIAA1429, one of the important components of methyltransferase complex, has been shown to promote cell growth and progression in various cancer types, including breast cancer, liver cancer, gastric cancer, head, and neck squamous cell carcinoma, and testicular germ cell tumors [18–22]. KIAA1429 was highly expressed in breast cancer tissues, and the overall survival of breast cancer patients with high-expression KIAA1429 was significantly shorter than those with low-expression KIAA1429 [18]. In addition, KIAA1429 was considerably upregulated in hepatocellular carcinoma (HCC) tissues, and high expression of KIAA1429 was associated with poor prognosis among HCC patients [19]. Furthermore, KIAA1429 was upregulated in gastric cancer tissues and acted as an oncogene by stabilizing c-Jun mRNA in an m6A-independent manner. However, its role in colorectal cancer remains unclear.

Silencing information regulator 1 (SIRT1), a member of the HDAC family, is highly evolutionarily conserved histone and non-histone deacetylase. The expression of SIRT1 is positively correlated with tumor growth, chemo-resistance, and metastasis [23, 24]. In human nucleus pulposus cells, METTL14-dependent m6A methylation promoted the TNF-α-induced cell senescence via regulating SIRT1 mRNA level [25]. In addition, RNA m6A modification contributes to podocyte injury through posttranscriptional regulation of SIRT1 mRNA, which suggested SIRT1 mRNA as a target of m6A methyltransferase [26]. In the present study, we confirmed the function of KIAA1429 in regulating colorectal cancer cells growth in vitro and in vivo and explored the underlying molecular mechanism, which provided a potential therapeutic target for colorectal cancer.

## Results

### The expression of KIAA1429 is increased in human colorectal cancer cells

**Fig. 1:**
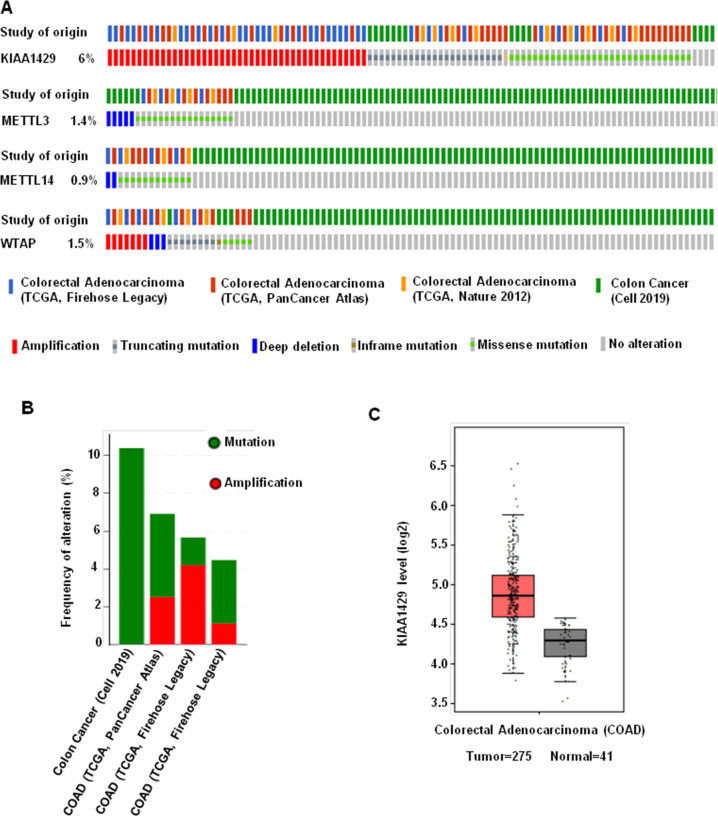
The expression of KIAA1429 in colorectal cancer. **A**, **B** TCGA database showed different gene alteration frequencies of KIAA1429 in colorectal cancer. **C** Expression level of KIAA1429 in tumor is higher than in normal tissue, shown by the GEPIA database.

KIAA1429 was shown to play important role in various cancers, including gastric cancer, lung cancer, and hepatocellular carcinoma. To explore the potential function of KIAA1429 in colorectal cancer, we analyzed the publicly available databases TCGA (The Cancer Genome Atlas) and GEPIA (Gene Expression Profiling Interactive Analysis). The TCGA data indicated that KIAA1429 showed highest amplification ratio, around 6%, in colorectal cancer among m^6^A methyltransferases (Fig. 1A). Also, the ratio of colorectal cancer patients with KIAA1429 genetic alterations could reach above 10% (Fig. 1B). In addition, the data from GEPIA showed that the expression level of KIAA1429 in tumor (*n* = 275) is higher than in normal tissue (*n* = 41) (Fig. 1C).

**Fig. 2:**
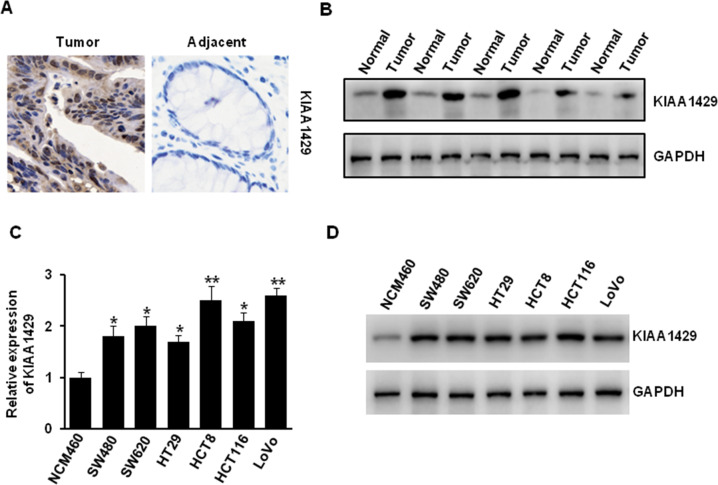
KIAA1429 was upregulated in colorectal cancer cells. **A** IHC analysis of KIAA1429 in colorectal adenocarcinomas and adjacent tissues. **B** The protein level of KIAA1429 in human colorectal adenocarcinomas tissues and adjacent normal tissues. **C**, **D** The mRNA and protein level of KIAA1429 in human normal colon mucosal epithelial cell and colorectal cancer cell lines was detected by QRT-PCR (**C**) and western blotting (**D**). *P < 0.05, **P < 0.01.

To further determine the expression of KIAA1429 in colorectal cancer, we performed immunohistochemistry analysis of KIAA1429 in colorectal cancer tissues and paired non-tumor tissues. The positive expression of KIAA1429 is more frequent, and the representative images are shown in Fig. 2A. In addition, our western blotting experiment also indicated that the protein level of KIAA1429 in tumor tissue was higher than that in adjacent normal tissues (Fig. 2B). Furthermore, we compared the mRNA and protein expression of KIAA1429 in normal human colon mucosal epithelial cell line (NCM460) and colorectal cancer cell lines (SW480, SW620, HT29, HCT8, HCT116, and LoVo). The results indicated that KIAA1429 was highly expressed in colorectal cancer cells compared with normal colon mucosal epithelial cells (Fig. 2C, D). All these data suggested that KIAA1429 was highly expressed in colorectal cancer cell.

### KIAA1429 knockdown inhibited colorectal cancer cells proliferation, colony formation, and motility

**Fig. 3:**
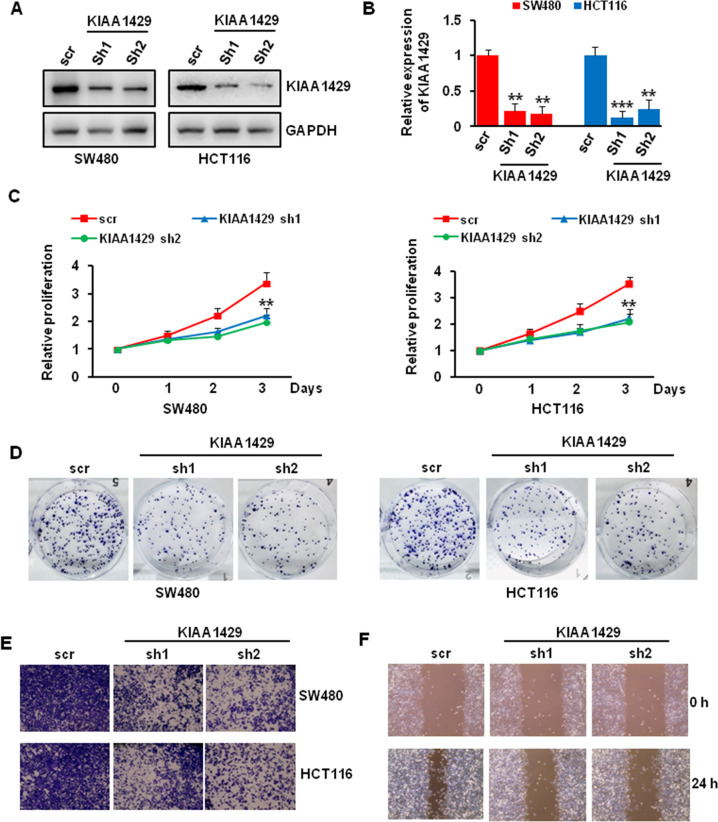
Effect of KIAA1429 depletion on cells proliferation, colony formation, and migration. **A**, **B** The knockdown efficiency of KIAA1429 in colorectal cancer cells was detected by western blotting (**A**) and QRT-PCR (**B**). **P < 0.01, ***P < 0.001. **C** The proliferation of SW480 and HCT116 cells after KIAA1429 depletion over 3 days was measured using CCK-8 assay. **P < 0.01. **D** The growth of SW480 and HCT116 cells over 14 days after KIAA1429 knockdown was measured using colony formation assay. **E** Transwell migration assay of SW480 and HCT116 cells after KIAA1429 knockdown. **F** Wound healing assay of SW480 cells after KIAA1429 knockdown.

To investigate the function of KIAA1429 in regulating colorectal cancer cell growth, we used specific shRNA to knockdown KIAA1429 in SW480 and HCT116 cells. The knockdown efficiency was presented in Fig. 3A, B, about 80% KIAA1429 was knockdown in both cell lines. We then sought to know the effect of KIAA1429 depletion on colorectal cancer cell growth. Our CCK8 results indicated that KIAA1429 knockdown significantly repressed SW480 and HCT116 cells proliferation (Fig. 3C). Consistent with the proliferation assay, our colony-forming unit assay also showed that KIAA1429 depletion inhibited the colony formation ability of SW480 and HCT116 cells (Fig. 3D). To determine whether KIAA1429 regulates cell motility, we performed migration in SW480 and HCT116 cells and wound healing assay in SW480 cells. As shown in Fig. 3E, F, the migration and wound healing ability was greatly inhibited in KIAA1429 depleted cells compared with scr control infected cells.

### KIAA1429 overexpression promoted colorectal cancer cells proliferation, colony formation, migration, and wound healing

**Fig. 4:**
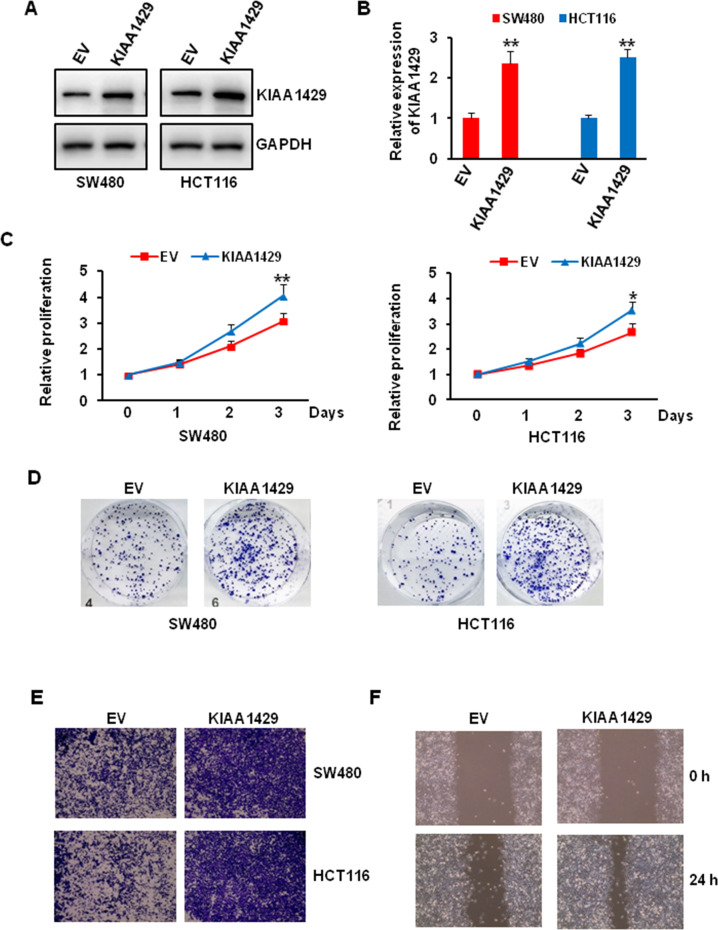
Effect of KIAA1429 overexpression on cells proliferation, colony formation, and migration. **A**, **B** The overexpression efficiency of KIAA1429 in colorectal cancer cells was detected by western blotting (**A**) and QRT-PCR (**B**). **P < 0.01. **C** The proliferation of SW480 and HCT116 cells after KIAA1429 overexpression over 3 days was measured using CCK-8 assay. *P < 0.05, **P < 0.01. **D** The growth of SW480 and HCT116 cells over 14 days after KIAA1429 overexpression was measured using colony formation assay. **E** Transwell migration assay of SW480 and HCT116 cells after KIAA1429 overexpression. **F** Wound healing assay of SW480 cells after KIAA1429 overexpression.

Since KIAA1429 knockdown significantly affected colorectal cancer cell biology, we next wondered whether overexpression of KIAA1429 has the reverse results. We first transfected the SW480 and HCT116 cells with empty or KIAA1429 plasmid, about two times level KIAA1429 was observed compared with empty vector (Fig. 4A, B). Our proliferation assay and colony-forming unit assay also showed that KIAA1429 overexpression increased the growth of SW480 and HCT116 cells (Fig. 4C, D). To determine the function of KIAA1429 in regulating cell motility, we performed migration in SW480 and HCT116 cells and wound healing assay in SW480, the migration and wound healing ability was greatly increased in KIAA1429 overexpressing cells compared with empty control transfected cells (Fig. 4E, F).

### KIAA1429 regulated colorectal cancer cells growth via SIRT1

**Fig. 5:**
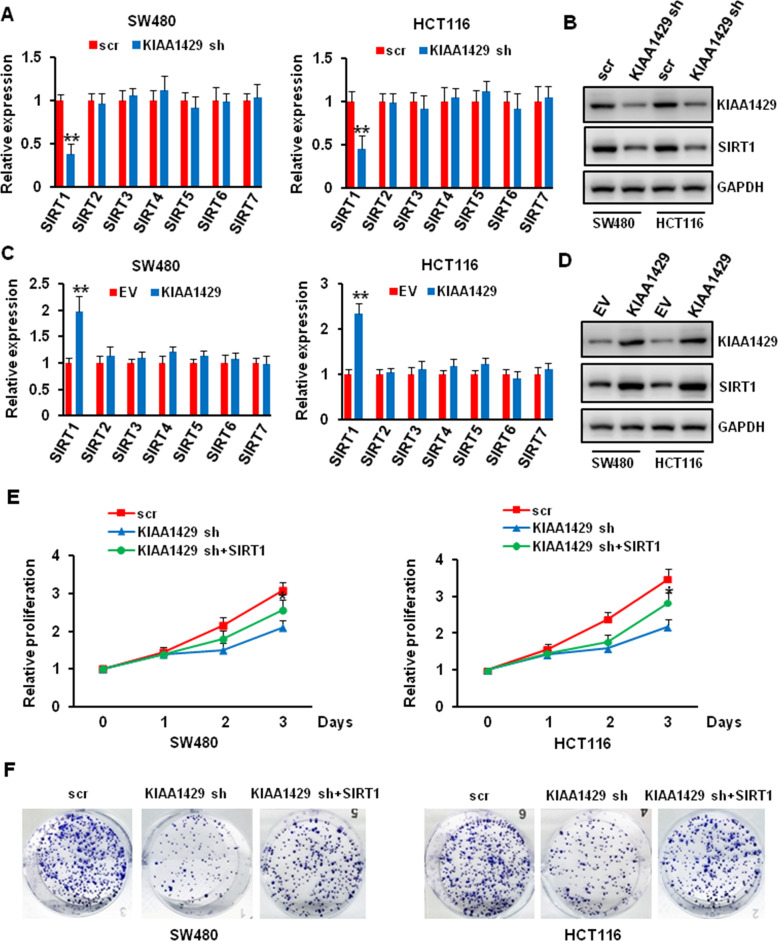
Effect of KIAA1429 on regulating the expression of SIRT1. **A**, **B** SW480 and HCT116 cells were infected with scr and KIAA1429 shRNA, the mRNA and protein level of SIRTs were detected by QRT-PCR (**A**) and western blotting (**B**). **P < 0.01. **C**, **D** SW480 and HCT116 cells were transfected with empty vector (EV) and KIAA1429 plasmid, the mRNA and protein level of SIRTs were detected by QRT-PCR (**C**) and western blotting (**D**). **P < 0.01. **E** The proliferation of SW480 and HCT116 cells after KIAA1429 depletion followed with or without SIRT1 overexpression was measured using CCK- 8 assay. *P < 0.05. **F** The growth of SW480 and HCT116 cells over 14 days after KIAA1429 depletion followed with or without SIRT1 overexpression was measured using colony formation assay.

Although we proved that KIAA1429 played important role in colorectal cancer cell biology, the mechanism remains unclear. Sirtuins (SIRTs) are class III histone deacetylases (HDACs), and accumulating evidence shows that SIRTs play contradictory roles in various malignancies, including colorectal cancer. We wonder whether KIAA1429 could regulate the expression of SIRTs. Thus, we knockdown and overexpressed KIAA1429 in SW480 and HCT116 cells, and evaluated the expression of SIRTs. As shown in Fig. 5A–D, KIAA1429 depletion or overexpression significantly decreased or increased the expression of SIRT1, but not other SIRTs. To further confirm that KIAA1429 regulated the growth of colorectal cancer cell via SIRT1, we knockdown KIAA1429 and then overexpressed SIRT1. The overexpression of SIRT1 significantly reversed the effect of KIAA1429 depletion on cell proliferation and increased the colony formation ability (Fig. 5E, F). These data indicated that KIAA1429 regulated colorectal cancer cells growth via SIRT1.

### KIAA1429 regulated the mRNA stability of SIRT1

**Fig. 6:**
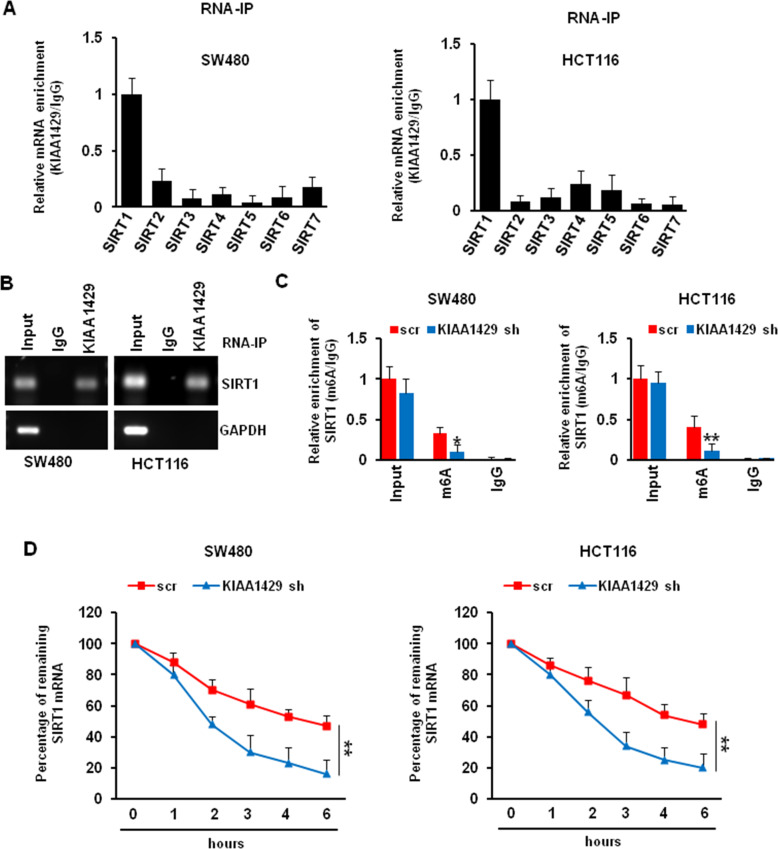
Effect of KIAA1429 on regulating SIRT1 mRNA stability. **A** SW480 and HCT116 cell lysates were immunoprecipitated with KIAA1429 antibody followed by QRT-PCR measuring transcript levels of all indicated targets. **B** PCR measured transcript levels of SIRT1 and GAPDH within KIAA1429 or IgG immunocomplexes in SW480 and HCT116 cell lysates. **C** SW480 and HCT116 cells were infected with scr and KIAA1429 shRNA, the cell lysates were immunoprecipitated with m^6^A antibody followed by QRT-PCR measuring transcript levels of SIRT1. *P < 0.05, **P < 0.01. **D** SW480 and HCT116 cells were infected with scr and KIAA1429 shRNA for 72 h, then treated with 8 μg/ml Act D for 0, 1, 2, 4, 6, 8 h. The stability of SIRT1 mRNA was described in Materials and Methods. **P < 0.01.

To investigate the mechanism of how KIAA1429 regulates the expression of SIRT1, we performed an RNA immunoprecipitation (RIP) assay to evaluate the interaction between KIAA1429 and SIRTs mRNA. As shown in Fig. 6A, SIRT1 mRNA was mostly associated with KIAA1429 protein in both SW480 and HCT116 cells. This result was further confirmed by RT-PCR indicating that SIRT1 transcript was present in KIAA1429 immunocomplex, but not in the control IgG immunocomplex. KIAA1429 was unable to bind to GAPDH mRNA, which served as a negative control (Fig. 6B). Since KIAA1429 is an important methyltransferase that participates in m6A modification, we wonder whether KIAA1429 depletion would alter the m6A modification on SIRT1 transcript. Thus, we used anti-m6A antibody to perform RIP, and we found that KIAA1429 knockdown significantly decrease the amount of SIRT1 mRNA modified by m6A in SW480 and HCT116 cells (Fig. 6C). m6A modification relates to the stability of mRNA. After KIAA1429 knockdown, the half-life of SIRT1 mRNA was decreased from 4.2 h to 1.9 h in SW480 cells. Similarly, the half-life of SIRT1 mRNA was decreased from 4.6 to 2.5 h in HCT116 cells (Fig. 6D). These suggested that KIAA1429 regulated the SIRT1 mRNA stability in an m6A-dependent manner.

### KIAA1429 depletion inhibited colorectal tumor growth in vivo

**Fig. 7:**
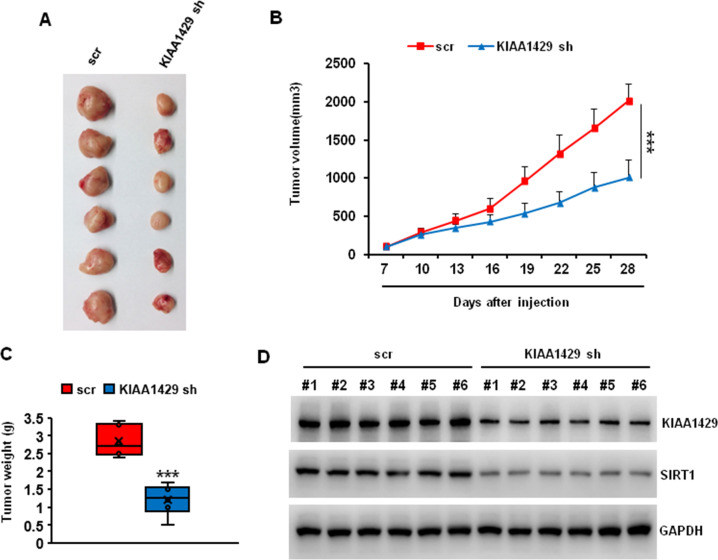
Effects of METTL3 depletion on tumor growth in vivo. **A** Typical photos of tumors on day 28 from scr and KIAA1429 shRNA groups. **B**, **C** KIAA1429 depletion significantly decreased the tumor size (**B**) and weight (**C**). ***P < 0.001. **D** Protein levels of KIAA1429 and SIRT1 in six representative tumors from each group were analyzed by western blotting.

To evaluate the possibility of KIAA1429 as a therapeutic target in colorectal cancer, we tested the function of KIAA1429 on tumor growth in a mouse xenograft model. We subcutaneously injected SW480 cells that transduced with Scr or KIAA1429 shRNA into NOD/SCID mice frank. The tumor size was measured every three days. At the end of the experiments (4 weeks), the mice were euthanized and the tumor weight was analyzed. As shown in Fig. 7A–C, the tumor size and weight were significantly decreased in KIAA1429 depletion group compared with control group. To assess the effect of KIAA1429 depletion on SIRT1, six representative tumors from each group were analyzed using western blotting. The protein level of KIAA1429 and SIRT1 was significantly lower in tumors with KIAA1429 depletion (Fig. 7D). These data indicated that KIAA1429 regulated tumor growth in vivo.

## Discussion

Increasing evidence has indicated that m^6^A key enzymes play critical role in human cancers, especially colorectal cancer. METTL3 is significantly upregulated in colorectal cancer tissues, and promotes its malignancy via stabilizing Snail mRNA [27]. In addition, METTL3 is reported to maintain SOX2 expression in an m^6^A-dependent mechanism to facilitate the progression of colorectal adenocarcinoma [28]. m^6^A mRNA methylation of DEGS2, a key ceramide-synthesizing enzyme, regulates the Cer metabolism to control cell proliferation in colorectal cancer [29]. Furthermore, FTO functioned as a tumor suppressor in colorectal cancer by inhibiting metastasis-associated protein 1 (MTA1) expression in an m^6^A-dependent manner [30].

As the “largest” component of methyltransferase complex, KIAA1429, was reported to be involved in the entire process of mRNA methylation in human cells, and a fourfold decrease in m6A peak scores has been observed after KIAA1429 knockdown, which is more than METTL3 and METTL14 knockdown [11]. In addition, KIAA1429 could recruit other m^6^A methyltransferase components, such as METTL3, METTL14, and WTAP, to guide region-selective methylations [31], which suggests the critical role of KIAA1429 in m^6^A modification. In general, KIAA1429 has been reported to facilitate cancer progression and is associated with poor survival in various cancer types. In breast cancer, KIAA1429 played its oncogenic role by regulating CDK1 [18]. Also, KIAA1429 mediated the m^6^A methylation of GATA3 pre-mRNA, which caused altered GATA3 expression and malignant phenotypes of hepatoma cells [19]. Furthermore, KIAA1429 was upregulated in gastric cancer tissues, and promotes the proliferation of gastric cancer cells via regulating the c-Jun mRNA stability [20]. On the contrary, KIAA1429 was also reported to be downregulated, predicted a better prognosis, and act as a tumor suppressor in papillary thyroid carcinoma [32]. However, these results were only obtained from TCGA database, which need further confirmation. In this study, we first analyzed the data from TCGA and GEPIA database and found that KIAA1429 was highly expressed in colorectal cancer. We then confirmed these results in patients’ tissue and colorectal cancer cell lines. More importantly, the expression level of KIAA1429 was positively related to the cancer cell growth and migration. All of these data suggested that KIAA1429 acts as a oncogene in colorectal cancer.

Sirtuins (SIRT1–7), a family of proteins with homology with the silent information regulator 2 (Sir2) gene in *Saccharomyces cerevisiae*, are protein deacetylases that contain highly conserved enzymes categorized as Class III histone deacetylases (HDACs III) [33]. The subcellular locations of sirtuins are heterogeneous, and sirtuin proteins participate in a wide range of biological processes [34]. To date, the role of sirtuins in malignancy has attracted more and more attention, but it is still under debate. SIRT2 demonstrated an inhibitory role in cell growth and acted as a tumor suppressor in tumors, especially in colorectal cancer [35–37]. SIRT4 was also reported as a tumor suppressor in colorectal cancer [38, 39]. Unlike SIRT2 and SIRT4, in the colorectal cancer, SIRT1 was reported to participate in the tumorigenesis, and its expression is positively correlated with the cancer progression in clinic [40–42]. Here, we found that KIAA1429 regulated the expression of SIRT1, but not other sirtuins. More interestingly, the inhibitory effect of KIAA1429 knockdown was reversed by SIRT1 overexpression, which indicated that KIAA1429 regulated cell growth via SIRT1. KIAA1429 can affect cancer progression in an m^6^A-dependent manner or m^6^A-independent manner. In liver cancer, KIAA1429 regulated the expression of GATA3 in an m^6^A-dependent manner [19], while KIAA1429 played its oncogenic role in breast cancer by regulating CDK1 in an m^6^A-independent manner [18]. In order to investigate the mechanism of how KIAA1429 regulates the expression of SIRT1, we performed an RIP assay, and found that only SIRT1 transcripts could be immunoprecipitated by KIAA1429. More importantly, the m^6^A modification of SIRT1 mRNA was significantly decreased after KIAA1429 depletion. Since m^6^A modification relates to the stability and splicing of mRNA, we then assessed the stability of SIRT1 mRNA. Not surprisingly, the stability of KIAA1429 mRNA was also significantly decreased after KIAA1429 depletion.

Taken all together, in the present study, we determined the important role of KIAA1429 in regulating colorectal cancer growth in vitro and in vivo. Mechanistically, KIAA1429 stabilized the SIRT1 mRNA in an m^6^A-dependent manner, which consequently promotes tumor progression. Our results highlight the functional role of KIAA1429 as a potential prognostic biomarker and therapeutic target in colorectal cancer.

## Materials and methods

### Clinical samples

CRC tumor and adjacent normal tissues were obtained from five patients (for protein extraction), all resected from August 2018 to August 2020. All tumors were verified as adenocarcinomas. The use of clinical samples was approved by the Human Research Ethics Committee of the Xiangya Hospital of Central South University.

### Antibodies

Antibodies against SIRT1 (#8469), and GAPDH (#5174) were obtained from Cell Signaling (MA, USA); antibody against KIAA1429 (#PA5-95717), m^6^A (#MA5-33030) for RNA-IP was purchased from ThermoFisher Scientific (MA, USA).

### Cell culture

Human NCM460 colonocytes were purchased from INCELL (TX, USA) and cultured in Dulbecco’s Modified Eagle Medium (DMEM) supplemented with 10% (v/v) FBS and penicillin/streptomycin. The human colorectal cancer cell lines SW480, SW620, HT29, HCT8, HCT116, and LoVo were purchased from the American Type Culture Collection (ATCC, Manassas, VA, USA) and cultured in DMEM/F12 medium (Lonza, Basel, Switzerland) supplemented with 10% (v/v) FBS and penicillin/streptomycin.

### Short hairpin RNA (shRNA) knockdown

KIAA1429 shRNAs were purchased from Sigma-Aldrich (MO, USA). shRNA plasmids were co-transfected with packaging constructs according to the manufacturer’s instruction to package the lentiviruses. SW480 and HCT116 cells were incubated with lentivirus for 72 h.

### Plasmid overexpression experiment

SIRT1 overexpression plasmid was purchased from Addgene [43]. Briefly, SW480 and HCT116 cells were plated onto 6-well plate, then transfected with 2 μg empty vector or SIRT1 plasmid using Lipofectamine 2000 (Invitrogen, MA, USA) according to the manufacturer’s instructions. The cells were harvested after transfection for 24–48 h.

### Cell proliferation assay

Cell proliferation was performed using the CCK8 kit following the manufacturer’s instruction (Jiangsu KeyGENBioTECH Corp., Ltd, China). Three thousand to five thousand colorectal cancer cells were seeded into each well of a 96-well plate after being infected with KIAA1429 shRNA or transfected with KIAA1429 plasmid. Ten microliter CCK8 reagent was added to each well at the indicated time and incubated at 37 °C for 2 h. The absorbance at 450 nm was recorded with a 96-well plate reader.

### Colony formation assay

Thousand colorectal cancer cells were seeded into each well of a 6-well plate after being infected with KIAA1429 shRNA or transfected with KIAA1429 plasmid and incubated at 37 °C for 10–14 days. The cells were then fixed, stained with 0.2% crystal violet, and imaged. Clones that consisted of at least 50 cells were considered as one colony.

### Migration assay

Migration assay was performed using 8 μm transwell inserts (Corning, NY, USA). 1 × 10^5^ colorectal cancer cells in 100 μl serum-free medium was added into the inserts, and the inserts were then placed in 24-well plate that fulfilled with 600 μl complete growth medium. After 24 h, the inserts were washed with PBS, fixed with 4% formaldehyde, and stained with 0.5% crystal violet. Five random fields were selected for image per filter-bottom surface.

### Wound healing assay

5 × 10^5^ SW480 cells were seeded in each well of a 6-well plate after being infected with KIAA1429 shRNA or transfected with KIAA1429 plasmid, and cells were scratched with a 1 ml pipette tip after confluent. After washed with PBS slightly, the images were captured by using a microscope equipped with a digital camera. The images were recorded again using the same microscope after 24 h.

### RNA isolation and quantitative reverse transcription PCR (QRT-PCR)

RNeasy Mini Kit (Qiagen, MD, USA) was used to isolate total RNA, and the cDNA was synthesized using script cDNA synthesis kit (Bio-Rad, CA, USA) following the manufacturer’s instructions. Quantitative PCR was performed using SYBR Premix Ex Taq II (TaKaRa, Japan) on CFX96 real-time PCR detection system (Bio-Rad, CA, USA) with the primers in Table 1. Values for each gene were normalized to the expression of GAPDH.

**Table 1:** Primers of QRT-PCR.

Gene	Forward	Reverse
KIAA1429	AAGTGCCCCTGTTTTCGATAG	ACCAGACCATCAGTATTCACCT
SIRT1	TAGACACGCTGGAACAGGTTGC	CTCCTCGTACAGCTTCACAGTC
SIRT2	CTGCGGAACTTATTCTCCCAGAC	CCACCAAACAGATGACTCTGCG
SIRT3	CCCTGGAAACTACAAGCCCAAC	GCAGAGGCAAAGGTTCCATGAG
SIRT4	GTGGATGCTTTGCACACCAAGG	GGTTCAGGACTTGGAAACGCTC
SIRT5	GTCCACACGAAACCAGATTTGCC	TCCTCTGAAGGTCGGAACACCA
SIRT6	TGGCAGTCTTCCAGTGTGGTGT	CGCTCTCAAAGGTGGTGTCGAA
SIRT7	TGGAGTGTGGACACTGCTTCAG	CCGTCACAGTTCTGAGACACCA
GAPDH	AATCCCATCACCATCTTCCAG	AAATGAGCCCCAGCCTTC

### Western blot analysis

The colorectal cancer cells were lysed using RIPA buffer (Invitrogen, MA, USA) supplemented with protease and phosphatase inhibitor cocktail (Sigma-Aldrich, MO, USA). Twenty microgram total proteins were subjected to SDS-PAGE separation, and then transferred to a nitrocellulose membrane. The membranes were incubated with the primary antibodies at 4 °C overnight after blocked with 5% milk for 1 h at room temperature. After washed three times with TBST, the membrane was incubated with secondary antibodies for 1 h at room temperature. The signal was detected using Super Signal West Pico Chemiluminescent Substrate (Thermo Scientific, MA, USA), and the chemiluminescence was detected by exposing the membrane using Mini Chemiluminescent Imaging and Analysis System (Sagecreation). The densitometric quantification was performed using Image J software. Data are reported as means ± SEM of values from three experiments.

### mRNA stability analysis

For mRNA stability analysis, SW480 and HCT116 cells infected with scramble or KIAA1429 shRNA for 72 h were harvested with or without 5 mM Actinomycin D treatment for the indicated time. One microgram total RNA was transcribed into cDNA using the script cDNA synthesis kit (Bio-Rad, USA), and the gene expression was analyzed using QRT-PCR.

### RNA immunoprecipitation (RIP)

RIP was conducted using a Magna RNA-binding Protein Immunoprecipitation Kit (Millipore, MA, USA) according to the instruction. Briefly, 50 million cells were lysed in 500 μl RIP lysis buffer before the lysate was incubated with 10 μg KIAA1429 or m6A antibody or IgG (4 °C with mixing, overnight). Thirty microliter Dynabeads Protein G was then added (4 °C with mixing, 6 h). After the digestion with proteinase K, the RNA was isolated using the RNeasy Mini Kit (Qiagen, MD, USA). The purified RNA was dissolved in 30 μl RNAse-free water for further QRT-PCR analysis.

### In vivo xenograft experiment

5.0 × 10^6^ SW480 cells (infected with scr or KIAA1429 shRNA) that suspended in 50 ul PBS and mixed with an equal volume of matrigel were subcutaneously injected in a 6-weeks-old male NOD/SCID (The Jackson Laboratory, Stock No: 001303) mice flank. We started measuring tumor size at the indicated times one week after injection. Tumor size was calculated by 0.5 × (long diameter) × (short diameter)^2^. After 4 weeks, the mice were euthanized and tumor weight was measured. All animal experiments were conducted according to the NIH Guide for the Care and Use of Laboratory Animals.

### Statistical analysis

Data were presented as mean ± SD from three independent experiments. *P*-value was determined using paired Student’s *t*-test, and a *P*-value < 0.05 was considered statistically significant.

## Supplementary information

Supplemental Material

## Data Availability

All data generated or analyzed during this study are included in this published article and its supplementary information files.
