# Supplementary material for: m6A methyltransferase KIAA1429 acts as an oncogenic factor in colorectal cancer by regulating SIRT1 in an m6A-dependent manner
Source: Cell Death Discov. 2022 Feb 25;8:83. doi: 10.1038/s41420-022-00878-w (PMC8881457; doi:10.1038/s41420-022-00878-w)

Figure 2B

GAPDH


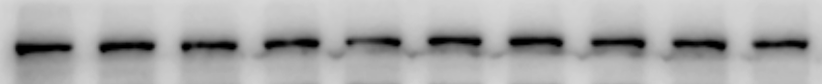


KIAA1429


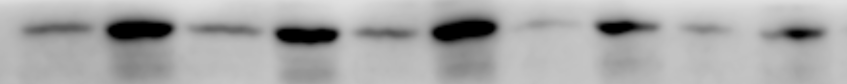


Figure 2D

GAPDH


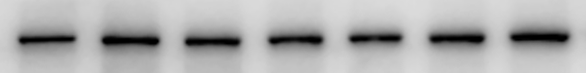


KIAA1429


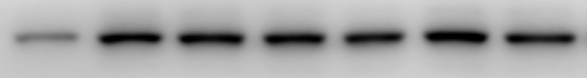


Figure 3A

GAPDH HCT116


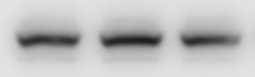


GAPDH SW480


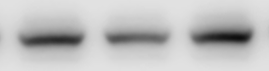


KIAA1429 HCT116


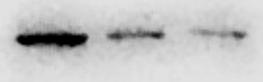


KIAA1428 SW480


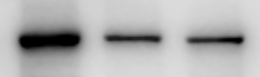


Figure 4A

GAPDH


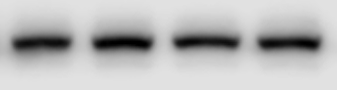


KIAA 1429


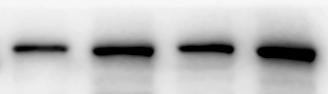


Figure 5B

GAPDH


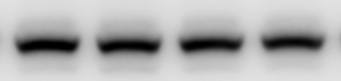


KIAA1429


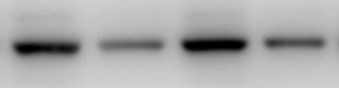


SIRT1


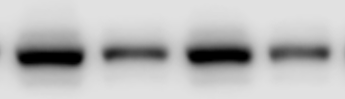


Figure 5D

GAPDH


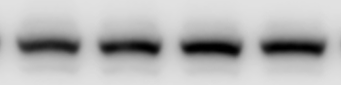


KIAA 1429


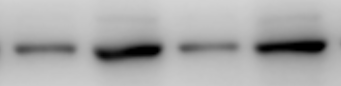


SIRT1


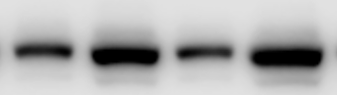


Figure 6B

GAPDH sw480


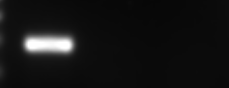


GAPDH hct116


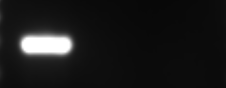


SIRT1 hct116


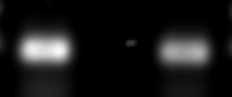


SIRT1 sw480


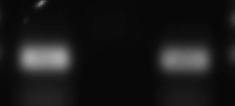


Figure 7D

GAPDH


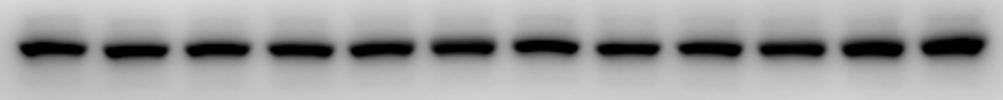


KIAA 1429


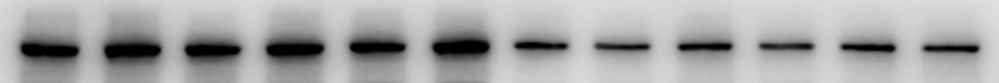


SIRT1


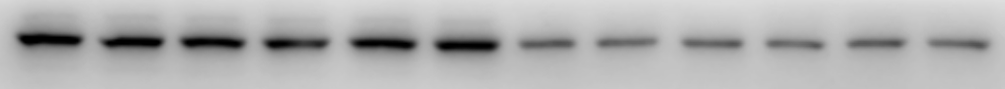

Supplement: Supplementary file 1 — Supplemental Material [file 41420_2022_878_MOESM1_ESM.docx]
